# Development and demonstration of a state model for the estimation of incidence of partly undetected chronic diseases

**DOI:** 10.1186/s12874-015-0094-y

**Published:** 2015-11-11

**Authors:** Ralph Brinks, Barbara H. Bardenheier, Annika Hoyer, Ji Lin, Sandra Landwehr, Edward W. Gregg

**Affiliations:** German Diabetes Center, Institute for Biometry and Epidemiology, Auf’m Hennekamp 65, Düsseldorf, 40225 Germany; Centers for Disease Control and Prevention, Division of Diabetes Translation, Atlanta, Georgia United States of America; University Hospital, Department for Statistics in Medicine, Düsseldorf, Germany

**Keywords:** Compartment model, Incidence, Prevalence, Diabetes, Chronic disease, Undiagnosed disease, Case finding, Screening, Health and Retirement Study

## Abstract

**Background:**

Estimation of incidence of the state of undiagnosed chronic disease provides a crucial missing link for the monitoring of chronic disease epidemics and determining the degree to which changes in prevalence are affected or biased by detection.

**Methods:**

We developed a four-part compartment model for undiagnosed cases of irreversible chronic diseases with a preclinical state that precedes the diagnosis. Applicability of the model is tested in a simulation study of a hypothetical chronic disease and using diabetes data from the *Health and Retirement Study (HRS)*.

**Results:**

A two dimensional system of partial differential equations forms the basis for estimating incidence of the undiagnosed and diagnosed disease states from the prevalence of the associated states. In the simulation study we reach very good agreement between the estimates and the true values. Application to the HRS data demonstrates practical relevance of the methods.

**Discussion:**

We have demonstrated the applicability of the modeling framework in a simulation study and in the analysis of the *Health and Retirement Study*. The model provides insight into the epidemiology of undiagnosed chronic diseases.

**Electronic supplementary material:**

The online version of this article (doi:10.1186/s12874-015-0094-y) contains supplementary material, which is available to authorized users.

## Background

Most major causes of chronic morbidity and mortality, including diabetes, cancer, osteoporosis, cardiovascular disease, and dementia, pass through undiagnosed stages, at which clinically defined and recognized thresholds for a particular disease have been met, but diagnosis has not occurred due to either lack of awareness, symptoms, or access to care [1–3]. In the case of diabetes, population surveys have shown that 24 % to 75 % of prevalent cases across different countries and settings have not been diagnosed and the diagnosis lag has been estimated as ranging from three to seven years [4, 5]. With regard to dementia, it is estimated that more than a half of all patients are undiagnosed [6].

High proportions or long durations of undiagnosed chronic disease have several important clinical and epidemiological ramifications. First, the period prior to diagnosis may be a missed opportunity to implement effective preventive interventions in clinical settings [7, 8]. Second, the undiagnosed state creates problems for the accurate monitoring of population health and response to public health interventions [9]. In the United States, for example, trends in diabetes incidence at a national level are assessed using self-reports of diagnosed cases [10]; this means that the degree to which recent diabetes trends have been influenced by shifting awareness or detection of existing cases, as opposed to the rate of occurrence of new cases of disease, is unclear.

**Fig. 1:**
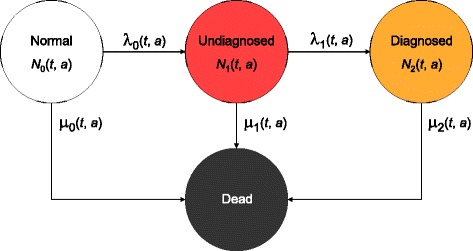
Chronic disease model with four states. Persons in the state *Normal* are healthy with respect to the disease under consideration. After onset of the disease, they change to state *Undiagnosed* and later to the state *Diagnosed*. The absorbing state *Dead* can be reached from all other states. The numbers of persons in the states and the transition rates depend on calendar time *t* and age a

Despite the importance of understanding the undiagnosed prevalence of chronic diseases, few methods have been considered to estimate rates of undiagnosed incidence in settings of incomplete data. Illness-death models have been developed to estimate incidence rates from prevalence data [11, 12]. Here we incorporate undiagnosed disease into an illness-death model using complementary information on prevalence and mortality, to permit estimation of undiagnosed incidence (Fig. 1). Estimation of incidence of undiagnosed chronic disease would provide a crucial missing link for the monitoring of chronic disease epidemics and for untangling the degree to which changes in prevalence are affected or biased by detection.

## Methods

Building upon previously published state models, for this study we develop a model including an *Undiagnosed* state (Fig. 1). The population of interest is partitioned into the four states *Normal* (i.e., healthy with respect to the chronic disease under consideration), *Undiagnosed, Diagnosed* (i.e., without and with a physician’s diagnosis), and *Dead*. The transition rates between the states are denoted as in the figure. The model described here is able to cope with secular trends, (i.e., involves calendar time *t*) and the different ages *a* of the subjects in the population, and thus these models are called *age-structured* [13].

The proportion of the living population in the states *Normal, Undiagnosed,* and *Diagnosed* are determined by their initial values and the rates *λ*_*ℓ*_,*μ*_*k*_, *ℓ*=0,1,*k*=0,1,2. Let *N*_0_,*N*_1_, and *N*_2_ denote the numbers of persons in the respective state *Normal, Undiagnosed,* and *Diagnosed*. In addition, we set *N*(*t, a*):=*N*_0_(*t, a*)+*N*_1_(*t, a*)+*N*_2_(*t, a*). For (*t, a*) with *N*(*t, a*)>0 define the prevalences $p_{k}(\textit {t, a}) := \frac {N_{k}(\textit {t, a})}{N(\textit {t, a})}, ~k = 0, 1, 2.$ For example, *N*_1_(*t, a*) denotes the number of persons aged *a* at time *t* with the disease, but without a diagnosis.

After deriving the governing equations for the state model in Fig. 1, we study an example of how the prevalences *p*_*k*_, *k*=0,1,2, evolve if the rates *λ*_*ℓ*_, *ℓ*=0,1, and *μ*_*k*_, *k*=0,1,2, are known. As we know the rates (i.e., the “causes”) and want to calculate the prevalences (i.e., the “effects”) we call this problem the *forward problem*.

Then, we examine whether the rates *λ*_*ℓ*_, *ℓ*=0,1, can be estimated if the prevalences *p*_*k*_ and the mortality rates *μ*_*k*_, *k*=0,1,2, are known. We call this problem the *inverse problem*. The inverse problem is important in epidemiology, in which surveying the prevalences *p*_*k*_ is much easier than surveying the transition rates *λ*_*ℓ*_. For surveying prevalences, cross-sectional studies suffice, whereas examining rates requires lengthy follow-up studies. We propose two approaches to solve the inverse problem.

After this, we describe and validate the methods in a simulation study and apply it to U.S. nationally representative data from the *Health and Retirement Study* (HRS). The HRS is a nationally representative longitudinal biannual survey of individuals 50 years of age and older in the United States. The survey is sponsored by the National Institute on Ageing and performed by the Institute for Social Research at the University of Michigan. The Health Sciences Institutional Review Board at the University of Michigan approved the HRS study design. The data used for this analysis contain no unique personal identifiers and are publicly available (after application). Permission to use the HRS data was obtained from the University of Michigan (Survey Research Center, 426 Thompson Street, Ann Arbor, MI 48104).

All calculations for this work have been performed with the statistical software R (The R Foundation for Statistical Computing). The scripts for usage in R are provided as an additional zip-file.

## Results

### The governing equations

Analogously to Brinks and Landwehr, [14], we look for the numbers *N*_0_(*t, a*),*N*_1_(*t, a*) and *N*_2_(*t,a*) of healthy, undiagnosed, and diagnosed persons in terms of partial differential equations (PDEs), which can be derived from the disease model in Fig. 1. For the healthy persons, we get the following initial value problem of Cauchy type:

(1) $$ \begin{aligned} (\partial_{t} + \partial_{a}) \, N_{0}(t, a) & = - \left(\mu_{0}(t, a) + \lambda_{0}(t, a) \right) \, N_{0}(t, a) \\ N_{0}(t, 0) & = S_{0}(t). \end{aligned}  $$

Here *S*_0_(*t*) is the number of healthy newborns at calendar time *t*. Note that, in this work, we just consider diseases contracted after birth. The notation *∂*_*x*_ denotes the partial derivative with respect to *x*, *x*∈{*t, a*}.

Although the inclusion of the disease duration *d* is also possible [12], hereinafter it is assumed that none of the rates depend on *d*. Then, the numbers *N*_1_ and *N*_2_ of diseased persons without and with diagnosis, respectively, are described similarly:

(2) $${} \begin{aligned} (\partial_{t} + \partial_{a}) \, N_{1}(t, a) & = - \left(\mu_{1}(t, a) + \lambda_{1}(t, a) \right) \, N_{1}(t, a)\\&\quad + \lambda_{0}(t, a)\, N_{0}(t, a)\\ N_{1}(t, 0) & = 0. \end{aligned}  $$

(3) $${} \begin{aligned} (\partial_{t} + \partial_{a}) \, N_{2}(t, a) & = -\mu_{2}(t, a) \, N_{2}(t, a) + \lambda_{1}(t, a)\, N_{1}(t, a)\\ N_{2}(t, 0) & = 0. \end{aligned}  $$

### Prevalence, incidence and mortality

In epidemiological contexts, it has become common to quantify the prevalences *p*_*k*_ instead of the absolute numbers *N*_*k*_, *k*=0,1,2. We expressed Eqs. (2) and (3) in terms of prevalences *p*_1_ and *p*_2_. The prevalence *p*_0_ can be substituted by using the equation *p*_0_=1−*p*_1_−*p*_2_. In addition, often the mortality *μ*_0_ is unknown and the overall mortality (general mortality) *μ* is given. The overall mortality *μ* in the population may be written as

$$\mu = p_{0} \, \mu_{0} + p_{1} \, \mu_{1} + p_{2} \, \mu_{2}. $$

Then, the PDEs (2) and (3) can be reformulated as

(4) $$ \begin{aligned} (\partial_{t} + \partial_{a}) p_{1} &= - \left(\lambda_{0} + \lambda_{1} + \mu_{1} - \mu \right)\, p_{1} - \lambda_{0}\, p_{2} + \lambda_{0} \end{aligned}  $$

(5) $$ \begin{aligned} (\partial_{t} + \partial_{a}) p_{2} &= \lambda_{1} \, p_{1} - \left(\mu_{2} - \mu \right) \, p_{2}. \end{aligned}  $$

Together with the initial conditions *p*_1_(*t*,0)=*p*_2_(*t*,0)=0 for all *t*, the system (4) - (5) completely describes the dynamics of the disease in the considered population. Note that the system (4) - (5) does not explicitly depend on the mortality of the healthy subjects *μ*_0_, which is typically unknown. The remaining rates are either accessible by (specially designed) epidemiological studies (*λ*_0_,*λ*_1_,*μ*_1_,*μ*_2_) or by official vital statistics (*μ*).

### Relation to the conventional illness-death model

The conventional illness-death model [15] does not distinguish between an undiagnosed or diagnosed disease state. Thus, the conventional illness-death model considers the states *Undiagnosed* and *Diagnosed* to be pooled. If we define the prevalence *p* as the pooled prevalence *p*=*p*_1_+*p*_2_, the system (4) - (5) can be used to derive the following equation:

(6) $$ (\partial_{t} + \partial_{a}) p = \left(1 -p \right) \, \left[\lambda_{0} - \left(\mu - \mu_{0} \right) \right].  $$

This equation has been proven in [14] for the conventional illness-death model. Thus, the system (4) - (5) is consistent with the conventional illness-death model if we pool the *Undiagnosed* and *Diagnosed* states together.

### Detection ratio

Once we have calculated the transition rates *λ*_0_ and *λ*_1_ for the model in Fig. 1, we can calculate a measure that we call the age-specific *detection ratioDR*. For (*t, a*) with *λ*_0_(*t, a*)>0 define

(7) $$ DR(t, a) = \frac{\lambda_{1}(t, a)}{\lambda_{0}(t, a)}.  $$

The detection ratio is a rate ratio. In the context of survival analysis such a ratio is called a *hazard ratio* [16]. For a point in time *t*, it describes the (instantaneous) probability of detecting an undiagnosed person of age *a* in relation to the (instantaneous) probability of a healthy subject aged *a* entering the *Undiagnosed* state.

A low detection ratio *DR*(*t, a*) implies that *p*_1_(*t, a*) increases. More precisely: For (*t, a*) let be *λ*_0_(*t, a*)>0 and *p*_1_(*t, a*)>0, then for small time intervals *δ*>0, a detection ratio *DR*(*t,a*) with

$$\begin{array}{@{}rcl@{}} DR(t, a) < \underbrace{\frac{p_{0}(t, a)}{p_{1}(t, a)} + \frac{\mu(t, a) - \mu_{1}(t, a)}{\lambda_{0}(t, a)}}_{=: \Delta(t,a)}, \end{array} $$

implies *p*_1_(*t*+*δ*,*a*+*δ*)>*p*_1_(*t, a*). Vice versa, a high detection ratio *DR*(*t, a*)>*Δ*(*t,a*), implies *p*_1_(*t*+*δ*,*a*+*δ*)<*p*_1_(*t, a*). This follows from (*∂*_*t*_+*∂*_*a*_)*p*_1_=0 for *DR*=*Δ*, see Eq. (4).

### Simulation: forward problem

We use system (4) - (5) to describe a hypothetical irreversible disease, which is unknown until a specific point in time *t*^⋆^. At *t*^⋆^ the disease is detected and no longer unknown. This could happen by the discovery of a new pathogen or a novel diagnostic technique or by increased awareness, attention, or access to care. Henceforth, physicians start to look for the disease. As a consequence, after *t*^⋆^ the prevalence *p*_1_ of undetected cases decreases, whereas the prevalence *p*_2_ of detected cases increases. As an example, the general mortality *μ* is chosen as the (approximated) general mortality of the German male population from 1900 to 2010. For the approximation of the mortality, we make the following approach:

$$\begin{array}{@{}rcl@{}} \mu (t, a) = \exp \left(\beta_{0}(t) + \beta_{1}(t) \, a \right), \end{array} $$

with *β*_0_(*t*)=−7.078−0.02592 *t* and *β*_1_(*t*)=0.06401+2.455 10^−4^*t*. Calendar time *t* is counted in years since January 1st, 1900.

For simplicity, the mortality rates *μ*_*ℓ*_, *ℓ*=1,2, are assumed to be proportional to *μ*:*μ*_1_=3.5 *μ* and *μ*_2_=2.5 *μ*. The factor for *μ*_1_ is chosen to be larger than the one for *μ*_2_, because in contrast to persons in the *Diagnosed* state, persons in the *Undiagnosed* state cannot be treated for the disease. The magnitude of the factors is motivated by dementia [17].

The rate *λ*_0_ (Table 1) is the 1.5-fold of the age-specific incidence rate of dementia in German males [18]. Dementia serves as a demonstration for an important chronic disease. However, as we are mixing data from different sources in different populations, the example is hypothetical and inferences about the disease itself should be drawn very carefully.

**Table 1:** Age-specific incidence rates in the simulation

Age	Incidence *λ*_0_	Incidence *λ*_1_ in the year 75
(years)	(per 100 person-years)	(per 100 person-years)
≤62.5	0	0
67.5	0.45	3.3
72.5	1.05	7.8
77.5	2.55	18.8
82.5	4.50	33.3
87.5	7.80	57.6
92.5	11.40	84.2
97.5	14.85	109.7
≥100	16.80	124.1

Age-specific incidence rates *λ*_0_ and *λ*_1_. For *t*>75 the rate *λ*_1_ increases by 1 % annually for all ages

For year *t*=75, the rates *λ*_1_ are also shown in Table 1. We assume a secular trend in *λ*_1_, mimicking increasing awareness of the hypothetical disease. In the simulation, *λ*_1_ increases by 1 % per year for all ages *a*.

**Fig. 2:**
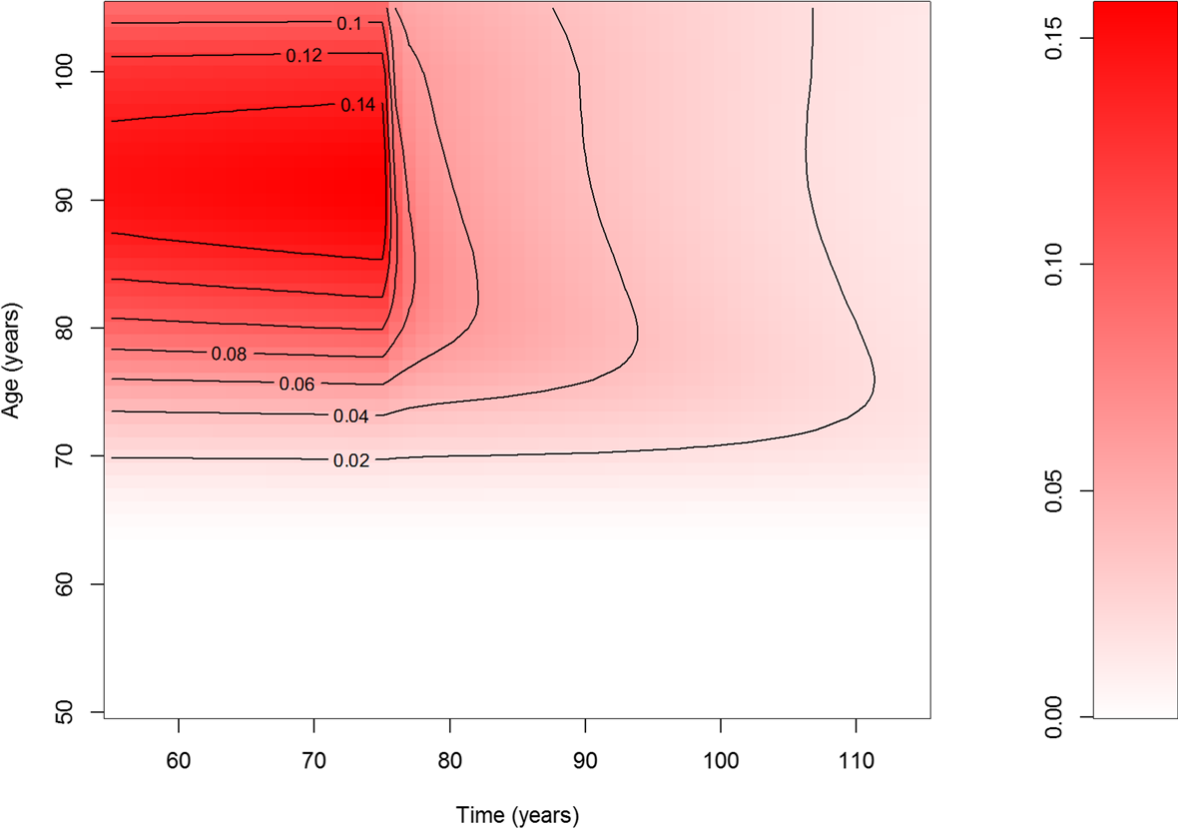
Prevalence of the undiagnosed disease. Prevalence *p*_1_ of undiagnosed disease over time *t* (*abscissa*) and age *a* (*ordinate*). The colour corresponds to value of the prevalence (coding scheme on the right part of the figure)

**Fig. 3:**
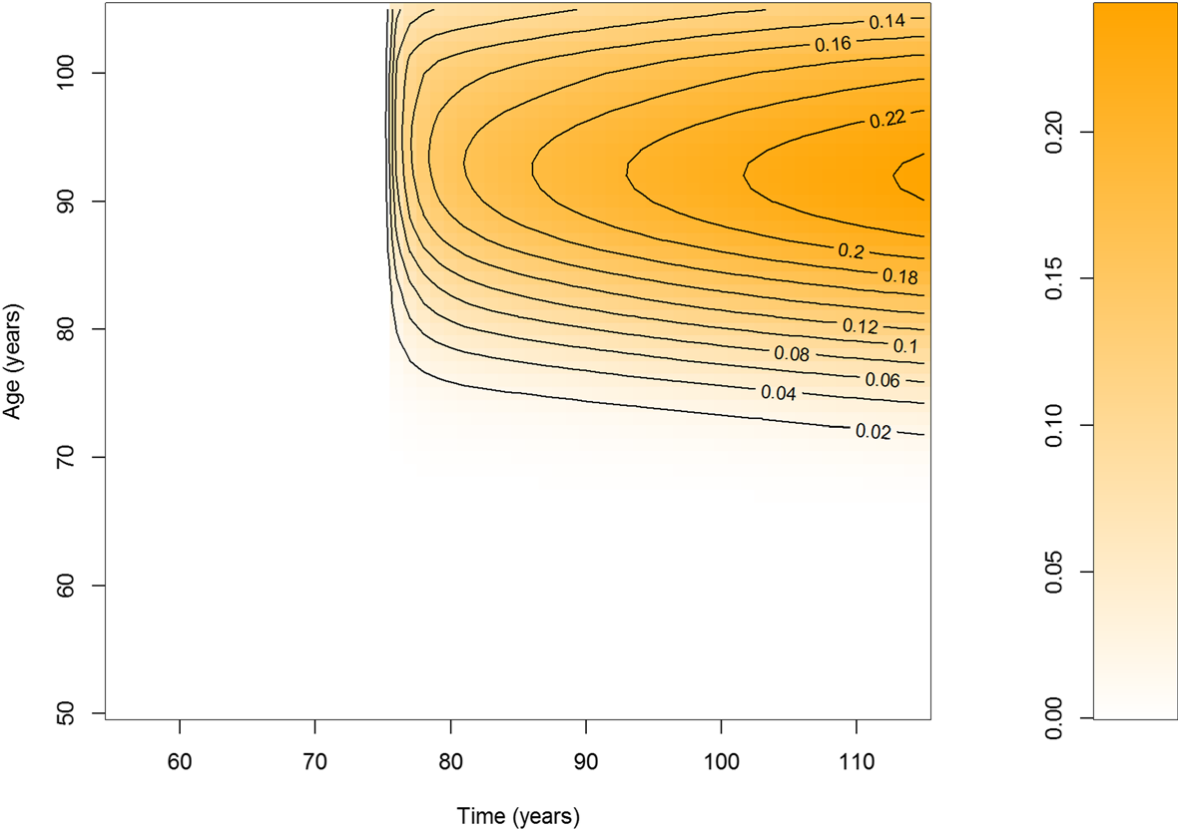
Prevalence of the diagnosed disease. Prevalence *p*_2_ of diagnosed disease over time *t* (*abscissa*) and age *a* (*ordinate*). The color corresponds to value of the prevalence (coding scheme on the right part of the figure)

If we solve the system (4) - (5) by the methods of characteristics [19], we obtain the prevalences of the undiagnosed and diagnosed disease as shown in Fig. 2 and 3, respectively. The qualitative change at *t*=75 in both prevalences *p*_1_ and *p*_2_ is clearly visible in the upper right corner of the figures.

**Fig. 4:**
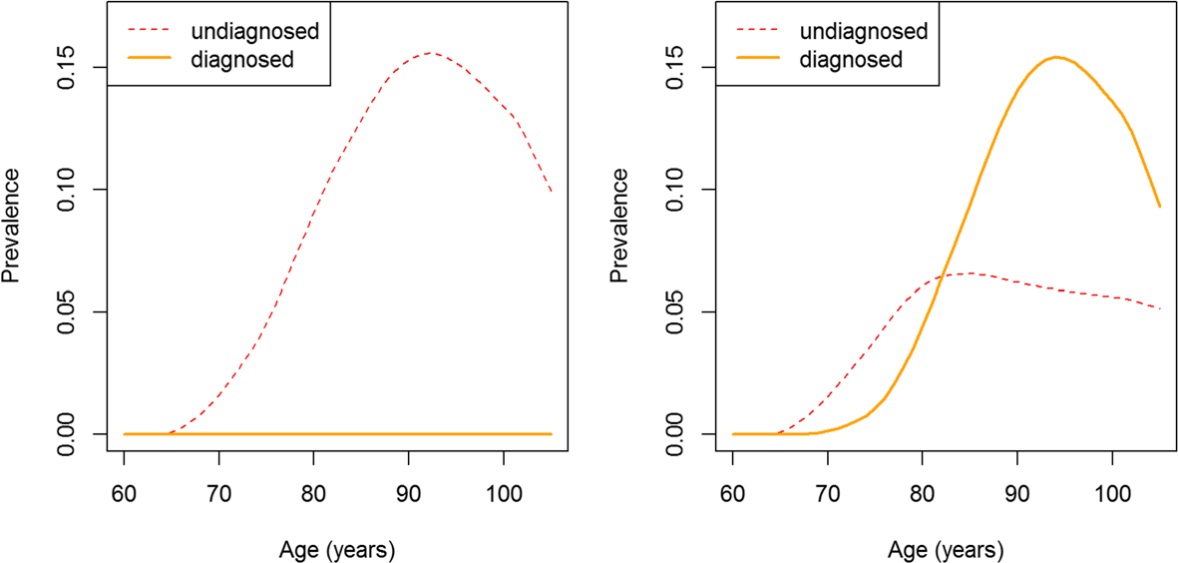
Prevalence of undiagnosed and diagnosed disease in years 70 and 80. Age-specific prevalence of undiagnosed (*red, dashed lines*) and diagnosed disease (*orange, solid lines*) in year *t*=70 (*left*) and in year *t*=80 (*right*)

For direct comparison, the age-specific prevalences in years *t*=70 and *t*=80 are additionally shown in Fig. 4. At *t*=70, there are no diagnosed cases (the hypothetical disease is not detected yet). The prevalence of the undiagnosed cases (*p*_1_) peaks at about 16 %, at the age of 91 years. Ten years later, the disease has been detected and the medical community is making diagnoses. Hence, the prevalence of the undiagnosed disease has decreased substantially - to less than 7 %. Especially in the higher age groups (≥85), physicians are aware of and detect a high proportion of cases and the prevalence of diagnosed cases (*p*_2_) has increased.

**Fig. 5:**
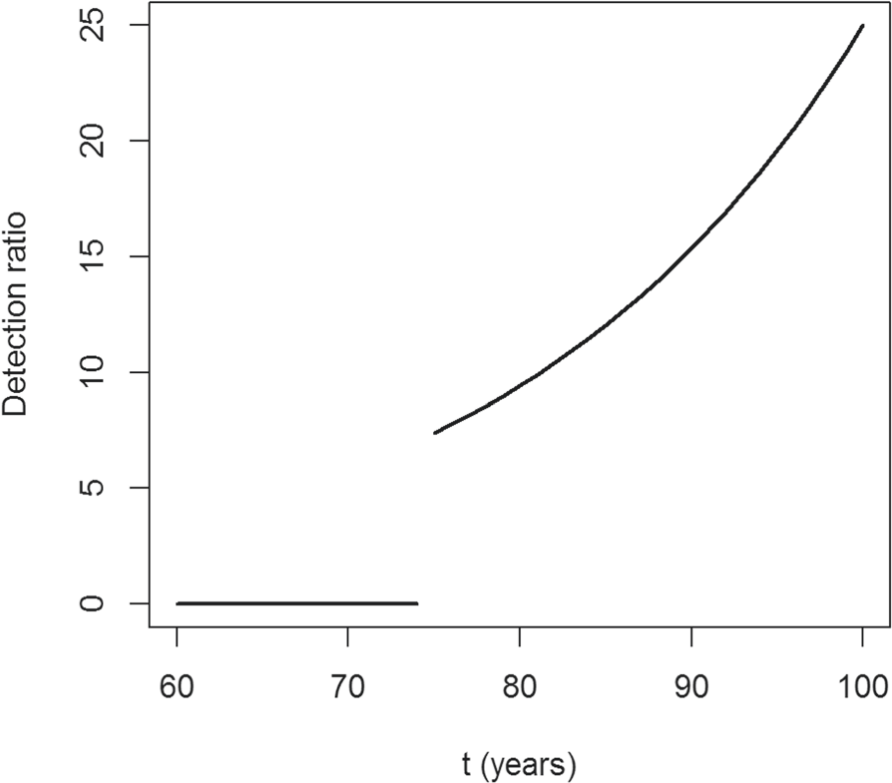
Detection ratio over calendar time. The detection ratio *DR* in the simulation depends only on the calendar time *t*, not on the age *a*. Before year *t*=75 the detection ratio *DR* is zero. After this the awareness for the hypothetical disease increases

In this example, the detection ratio $DR = \frac {\lambda _{1}}{\lambda _{0}}$ is chosen to be independent of the age *a*. It depends only on the calendar time *t*. The time course of *DR* is shown in Fig. 5. Before year 75, the detection ratio is 0. Later, the physicians start to diagnose the hypothetical disease at increasing rates.

**Fig. 6:**
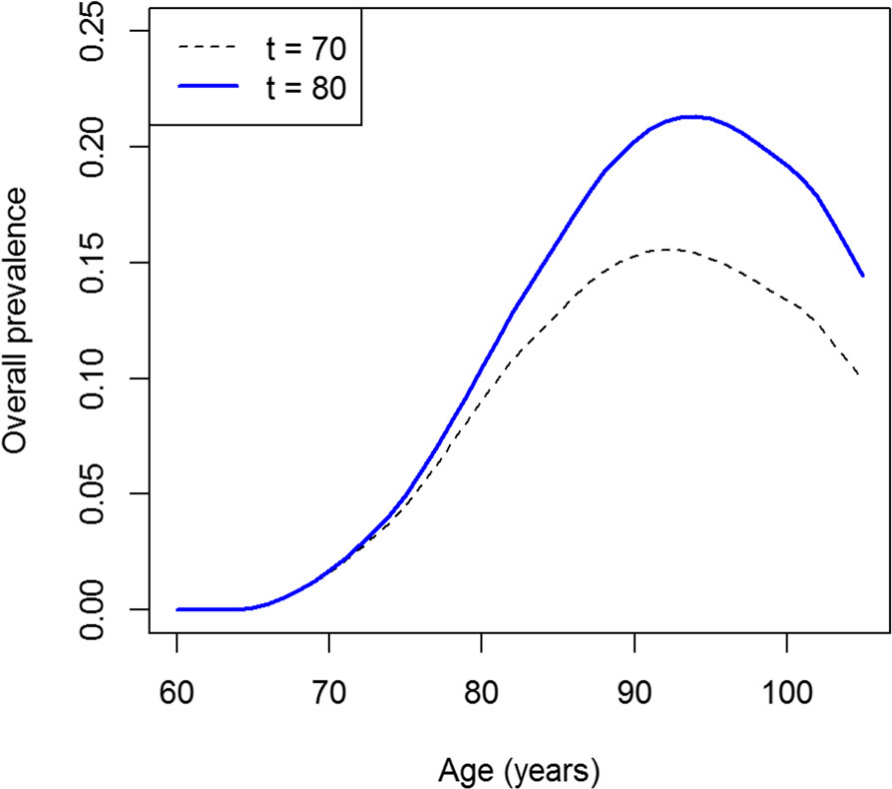
Overall prevalence of the disease in year 70 and 80. Age-specific overall prevalence (undiagnosed and diagnosed, *p*_1_+*p*_2_) in years *t*=70 (*black, dashed*) and *t*=80 (*blue, solid*)

The overall prevalence *p* (=*p*_1_+*p*_2_) in year *t*=70 differs substantially from the one at *t*=80 (Fig. 6), which is an effect of the lowered mortality for those diseased persons whose condition has been detected. As the mortality *μ*_2_ is considerably lower than *μ*_1_, the overall survival of the diseased persons is improved after *t*=75 and the overall prevalence increases.

### Inverse problem

An important epidemiological application is the calculation (of some) of the rates in the model, if the prevalences *p*_*k*_, *k*=1,2, are known. A typical situation might be that the mortality rates are recorded in death registries (or other vital statistics) and two cross-sectional surveys are conducted to obtain the age-specific prevalences *p*_*k*_, *k*=1,2, at two points in time, *t*_1_ and *t*_2_. The *inverse problem* is about whether the underlying rates *λ*_*ℓ*_, *ℓ*=0,1, can be *reconstructed* from the mortality and the prevalences. In the next two subsections we will present two ways for solving the inverse problem.

#### Direct solution of the inverse problem

We start with the observation, that Eq. (5) can be solved for *λ*_1_. For *p*_1_>0 it holds:

(8) $$ \lambda_{1} = \frac{(\partial_{t} + \partial_{a}) p_{2} + \left(\mu_{2} - \mu \right) \, p_{2}}{p_{1}}.  $$

With known *λ*_1_, Eq. (4) can be solved for *λ*_0_. For 1−*p*_1_−*p*_2_>0 it is:

(9) $$ \lambda_{0} = \frac{(\partial_{t} + \partial_{a}) p_{1} + \left(\lambda_{1} + \mu_{1} - \mu \right) \, p_{1}}{1- p_{1} - p_{2}}.  $$

This is the *direct solution* of the inverse problem.

To give a practical demonstration of the direct solution, assume that the age-specific prevalences *p*_*k*_, *k*=1,2, at two points in time *t*_*j*_, *j*=1,2, and the mortality rates *μ*,*μ*_1_, and *μ*_2_ are given at some time *t*^′^ with *t*_1_<*t*^′^<*t*_2_. Then we can approximate

(10) $${} {\fontsize{8.3pt}{9.6pt}\selectfont{\begin{aligned} (\partial_{t} + \partial_{a}) p_{k}(t', a) \doteq \frac{p_{k}(t_{2}, a + t_{2} - t') - p_{k}(t_{1}, a - t' + t_{1})}{t_{2} - t_{1}}, k=1,2. \end{aligned}}}  $$

The symbol $\doteq $ means that the partial derivative is approximated by its linearisation. Terms of quadratic or higher order in (*t*_2_−*t*_1_) are neglected.

**Fig. 7:**
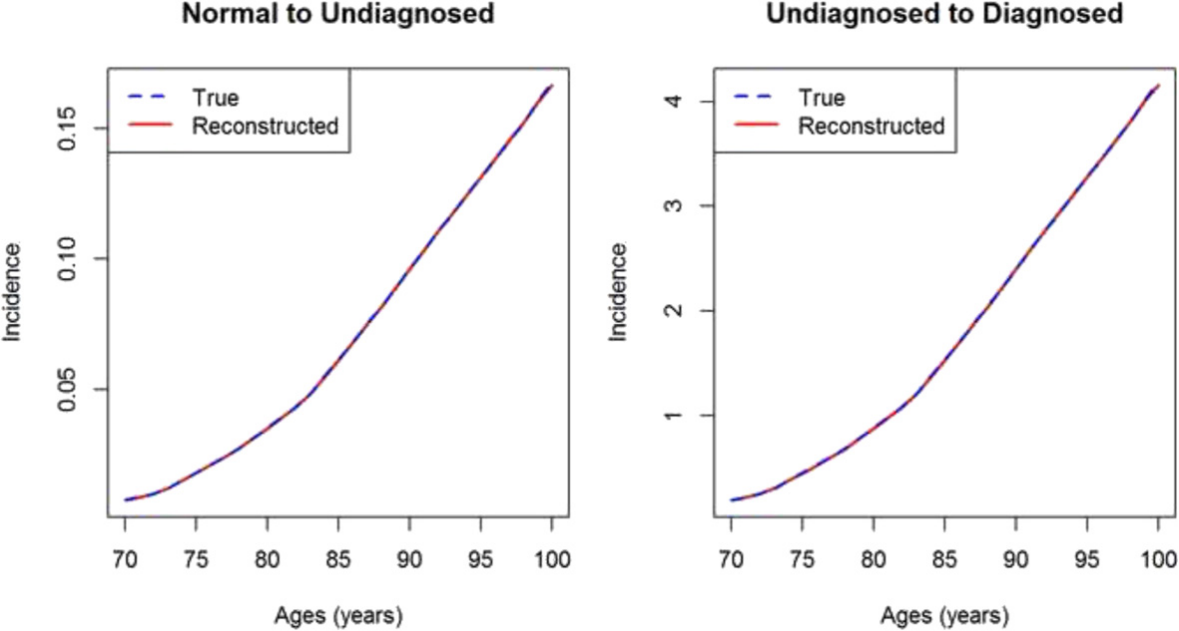
Direct solution of the inverse problem. The reconstructed (*red, solid*) and true rates (blue, dashed) *λ*_0_ (*left*) and *λ*_1_ (*right*). Visually the true and the reconstructed rates are indistinguishable

We show an application based on the data from the forward problem of the previous section. If we calculate *p*_1_ and *p*_2_ in *t*_1_=99 and *t*_2_=101 by solving the forward problem, and then apply Eqs. (8) and (9) by using the approximation in Eq. (10) for *t*^′^=100, we obtain the incidences *λ*_0_ and *λ*_1_ as shown Fig. 7. For comparison, the true incidences are shown as blue dashed lines. From visual inspection, the reconstructed incidences do not differ from the true incidences. Indeed, the maximum relative error in the age range *a*=70,71,…,100 is 0.90 % for *λ*_0_ and 1.26 % for *λ*_1_. The median relative errors are 0.13 % and 0.14 %.

#### Least squares solution

An alternative way of finding a solution for the inverse problem is given by the following approach. Assuming again we know the age-specific prevalences *p*_*k*_, *k*=1,2, at two points in time *t*_*j*_, *j*=1,2, and the mortality rates *μ*,*μ*_1_, and *μ*_2_ at some time *t*^′^ with *t*_1_<*t*^′^<*t*_2_. Typically, *p*_*k*_, *k*=1,2, are subject to sampling uncertainty. Let *σ*_*k*_ denote the standard error of *p*_*k*_. For a moment let us assume that we know *p*_*k*_ at *t*^′^, and that we have a “guess” $\lambda _{\ell }^{(g)}(t').$ Then, we can use the system (4) - (5) to approximate *p*_*k*_ at *t*_2_ by

(11) $${} {\fontsize{8.6pt}{9.6pt}\selectfont{\begin{aligned} p_{k} \left(t_{2}, a | \lambda_{\ell}^{(g)} \right) \doteq p_{k} \left(t', a - h_{2} \right) + h_{2} \, \left(\partial_{t} + \partial_{a} \right) p_{k} \left(t', a - h_{2} | \lambda_{\ell}^{(g)} \right) \end{aligned}}}  $$

in which *h*_2_=*t*_2_−*t*^′^. The values of the partial derivatives (*∂*_*t*_+*∂*_*a*_)*p*_*k*_ are calculated by the right-hand sides of the associated Eqs. (4) and (5), respectively.

Similarly, we may approximate *p*_*k*_ at *t*_1_:

(12) $${} {\fontsize{8.6pt}{9.6pt}\selectfont{\begin{aligned} p_{k} \left (t_{1}, a | \lambda_{\ell}^{(g)} \right) \doteq p_{k} \left(t', a - h_{1} \right) - h_{1} \, \left(\partial_{t} + \partial_{a} \right) p_{k}\left(t', a - h_{1} | \lambda_{\ell}^{(g)} \right) \end{aligned}}}  $$

in which *h*_1_=*t*^′^−*t*_1_.

As $\lambda _{\ell }^{(g)}$ was based on an arbitrary assumption that the calculated values $p_{k}(t_{j}, a | \lambda _{\ell }^{(g)}), ~k, j=1,2,$ are likely to deviate from the measured values *p*_*k*_(*t*_*j*_,*a*). Define the sum of standardized squared error $X^{2}(\lambda _{\ell }^{(g)})$ as

(13) $$ {\fontsize{9.2pt}{9.6pt}\selectfont{\begin{aligned} X^{2} \left(\lambda_{\ell}^{(g)} \right) &:= \frac{\| p_{1}(t_{1}, a) - p_{1}\left(t_{1}, a | \lambda_{\ell}^{(g)} \right) \|^{2}}{{\sigma^{2}_{1}}(t_{1}, a)} \, \\&\quad + \frac{\| p_{1}(t_{2}, a) - p_{1}\left(t_{2}, a | \lambda_{\ell}^{(g)} \right) \|^{2}}{{\sigma^{2}_{1}}(t_{2}, a)} \, \\ &\quad + \frac{\| p_{2}(t_{1}, a) - p_{2}\left(t_{1}, a | \lambda_{\ell}^{(g)} \right) \|^{2}}{{\sigma^{2}_{2}}(t_{1}, a)} \, \\&\quad+ \frac{\| p_{2}(t_{2}, a) - p_{2}\left(t_{2}, a | \lambda_{\ell}^{(g)} \right) \|^{2}}{{\sigma^{2}_{2}}(t_{2}, a)}. \end{aligned}}}  $$

Then, the inverse problem can be written as a minimization problem:

(14) $$ \lambda_{\ell} = \arg \min_{\lambda_{\ell}^{(g)} \ge 0} X^{2} \left (\lambda_{\ell}^{(g)} \right), ~\ell = 0, 1.  $$

Hence, *λ*_*ℓ*_ is the weighted least squares solution, which minimizes the squared deviation between the estimated and measured *p*_*k*_ in *t*_1_ and *t*_2_. Underlying the minimisation approach is the idea that the error $p_{k}(t_{j}, a) - p_{k}(t_{j}, a | \lambda _{\ell }^{(g)})$ is approximately normally distributed with mean 0 and standard deviation *σ*_*k*_(*t*_*j*_,*a*) [20].

So far, we have assumed that we know *p*_*k*_ at *t*^′^, which is not the case if we just have data from two cross-sections at *t*_1_ and *t*_2_. In this case, we can estimate *p*_*k*_(*t*^′^,*a*) by

$${} p_{k}(t', a) \doteq \frac{h_{2}}{h_{1}+h_{2}} p_{k}(t_{1}, a - h_{1}) + \frac{h_{1}}{h_{1}+h_{2}} p_{k}(t_{2}, a + h_{2}). $$

**Fig. 8:**
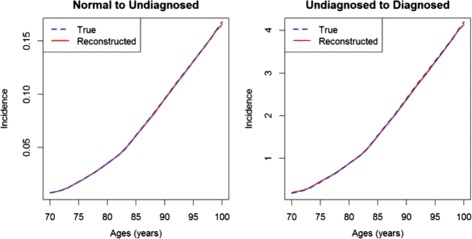
Least squares solution of the inverse problem. The reconstructed (*red, solid*) and true rates (*blue, dashed*) *λ*_0_ (*left*) and *λ*_1_ (*right*). Visually, the true and the reconstructed rates are nearly indistinguishable

We demonstrate the solution of the inverse problem by the least squares approach in the example above (see the previous section about directly solving the inverse problem). As we do not have sampling uncertainty in the example, we set *σ*_*k*_=1. For solving the (constraint) minimisation problem, we use the R package nloptr [21]. The result is shown in Fig. 8. The reconstructed incidences visually do not differ from the true incidences. The maximum relative error in the age range *a*=70,71,…,100 is 1.7 % for *λ*_0_ and 3.2 % for *λ*_1_. The median relative errors are 0.29 % and 0.67 %.

Compared to the direct solution the median and maximum relative error increases, which is a consequence of the approximations (11) and (12). However, the least squares approach allows the inclusion of an error model and an estimation of the resulting uncertainty in the *λ*_*ℓ*_ as shown in the next section.

### Example from the Health and Retirement Study

Estimates of diabetes prevalence and mortality were based on data from the 2006, 2008, and 2010 waves of the HRS.

Prevalence of undiagnosed diabetes was calculated from the random half sample of those participants aged 50 to 95 years, selected for the biomarkers blood tests in 2006 and 2008 [22] who had a valid HbA1c result (*n*=6300 and *n*=6115, respectively). Respondents who had a baseline HbA1c of >6.5 % and did not report a diabetes diagnosis were defined as having undiagnosed diabetes. Respondents in the 2006 sample (*n*=243) with undiagnosed diabetes were followed to 2008 to assess the risk of dying; similarly those in the 2008 sample (*n*=284) were followed to 2010. Due to the relatively low number of persons who died (*n*=19 and *n*=16), the mortality data of 2006 and 2008 have been pooled.

Diagnosed diabetes was identified if the respondent reported they had been told by a doctor that they had diabetes or high blood sugar [23]. Prevalence of diagnosed diabetes was based on 17,860 persons aged 50 to 95 years sampled in 2006 and 16,777 persons sampled in 2008. Respondents in the 2006 sample with diagnosed diabetes (*n*=3714) were followed to 2008 to assess mortality. During that time 408 subjects died. Similarly those in the 2008 sample (*n*=3768) were followed to 2010, with 503 death cases. For consistency reasons, we pooled the mortality data of both samples as the death cases in undiagnosed diabetes.

We used the survey sample in 2008 of participants aged 50 to 95 years who were alive, or who had died and had a proxy interview conducted by a family member or friend (*n*=17,970), to assess mortality (*n*=1173 died during the period 2008–10). To obtain mortality risk in the general population we ran a logistic model with death as the dependent variable and age and sex as the independent variables. Using the regression estimates (e.g., converting the odds to probabilities), we obtained the risk of mortality for each age, and then averaged every two years of age (i.e., 50–51, 52–53, …, 94–95).

**Fig. 9:**
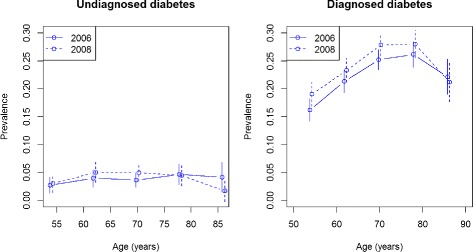
Prevalence of diagnosed and undiagnosed diabetes in men. Age course of the prevalence of undiagnosed (*left*) and diagnosed (*right*) diabetes in the male population of HRS in 2006 (*solid line*) and 2008 (*dashed line*). Vertical bars are 95 % confidence intervals

Figure 9 shows the age-specific prevalence of undiagnosed and diagnosed diabetes in the male population of the HRS in 2006 and 2008. In 2006 the prevalence of undiagnosed diabetes (*p*_1_, left part of Fig. 9) ranges from 3–4 %. Two years later this prevalence is about 5–6 %. Similarly, the prevalence of diagnosed diabetes (*p*_2_) has increased for all age groups (right part of Fig. 9).

**Fig. 10:**
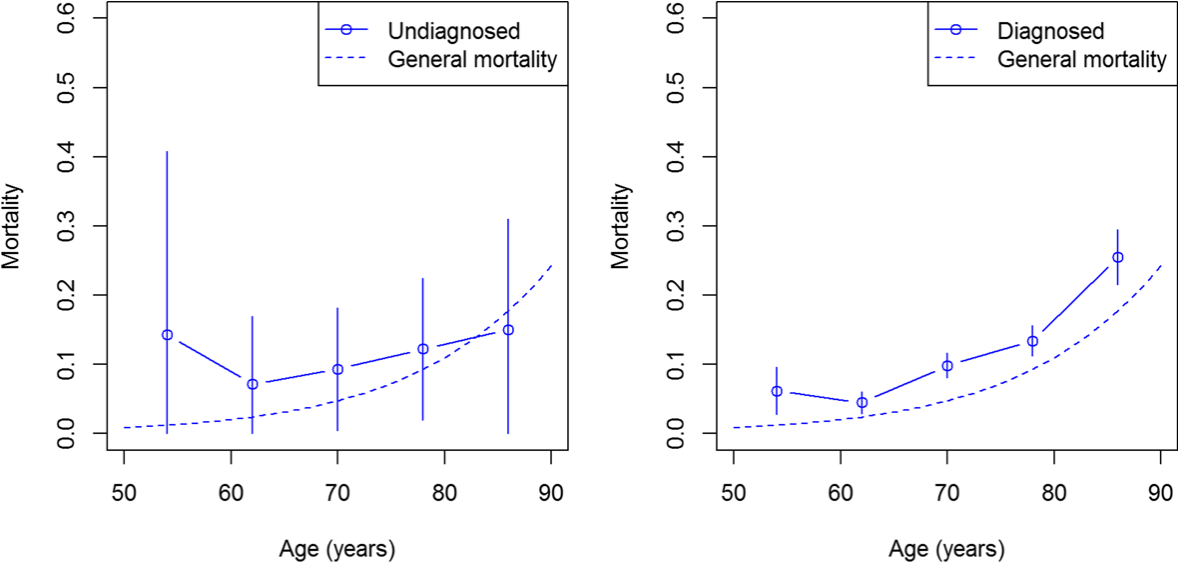
Mortality in men. Age course of the mortality in undiagnosed (*left*) and diagnosed (*right*) men of HRS from 2006 to 2008 (*solid line*). The dashed line is the general mortality. Vertical bars are 95 % confidence intervals

Among the prevalences, the mortality of the general population and the mortality of the undiagnosed and diagnosed subjects are needed as input data for the method. Figure 10 compares the risk of dying between 2006 and 2008 in the male HRS population (solid line) with the general population (dashed line).

After describing the input data for the method, we calculate the least squares solution, described in the previous section. For this Eq. (13) is slightly modified, because we need to estimate the probability of the death of a study participant. Therefore, *X*^2^ in Eq. (13) was augmented by the summand

$$\begin{array}{@{}rcl@{}} \frac{\| p_{m}(a) - p_{m}(a | R^{(g)}) \|^{2}}{{\sigma^{2}_{m}}(a)}, \end{array} $$

in which *p*_*m*_(*a*) is the observed age-specific mortality risk with standard deviation *σ*_*m*_. The modeled mortality risk *p*_*m*_(*a*|*R*^(*g*)^) is assumed be to proportional to the mortality risk *π*(*a*) of the general population, with *R*^(*g*)^ being the proportionality factor: *p*_*m*_(*a*|*R*^(*g*)^)=*R*^(*g*)^*π*(*a*).

To obtain estimates of the standard error of the *λ*_*ℓ*_, *ℓ*=0,1, we use a probabilistic sensitivity analysis [24]: 10,000 samples from the distributions of the input values are drawn and the associated least squares estimates for *λ*_*ℓ*_, *ℓ*=0,1, are calculated. This leads to an empirical estimate for the distribution of *λ*_*ℓ*_.

Tables 2 and 3 show the results of the reconstructed incidence rates *λ*_*ℓ*_, *ℓ*=0,1, for men and women. From the empirical standard deviations of the estimates it can be seen that the uncertainty in the estimates is rather high compared to the empirical mean. This is a result of the uncertainty in the input data, especially in the mortality of the persons with undiagnosed diabetes.

**Table 2:** Age-specific incidence rates for men in HRS

Age	Incidence *λ*_0_		Incidence *λ*_1_	
(years)	(per 100 person-years)		(per 100 person-years)	
	Mean	SD	Mean	SD
54	2.05	1.12	54.18	30.11
62	2.22	1.25	24.62	16.18
70	3.13	1.21	35.50	16.48
78	1.87	1.40	30.55	18.45
86	0.25	0.64	36.66	28.70

Age-specific incidence rates *λ*_0_ and *λ*_1_ for men as reconstructed from the prevalence and mortality data of the HRS study

**Table 3:** Age-specific incidence rates for women in HRS

Age	Incidence *λ*_0_		Incidence *λ*_1_	
(years)	(per 100 person-years)		(per 100 person-years)	
	Mean	SD	Mean	SD
54	1.41	0.88	25.39	16.00
62	2.77	0.96	42.51	19.20
70	2.60	1.02	19.07	12.07
78	1.53	1.11	21.28	12.97
86	1.46	1.32	7.85	9.13

Age-specific incidence rates *λ*_0_ and *λ*_1_ for women as reconstructed from the prevalence and mortality data of the HRS study

Due to the uncertainty in the estimated incidence rates, the corresponding age-specific detection ratios *DR* are estimated after performing a log-transformation. It holds that log(*DR*)= log*λ*_1_− log*λ*_0_. Thus, the variance of log(*DR*) can be estimated by the variances of log*λ*_*ℓ*_, *ℓ*=0,1, and the covariance. The corresponding estimates are shown in Table 4. We confine ourselves to reporting the estimates without interpreting them, because the uncertainty in estimated rates is too high to allow valid conclusions from these ratios.

**Table 4:** Logarithms of the age- and sex-specific detection ratios in HRS

Age (years)	Detection ratio			
	Men		Women	
	Mean	SD	Mean	SD
54	3.29	0.81	2.88	0.92
62	2.36	0.94	2.67	0.58
70	2.39	0.63	1.89	0.80
78	2.82	0.96	2.65	0.97
86	4.19	1.27	1.73	1.26

Logarithms of the age-specific detection ratios log*D* *R* for men and women in HRS

## Discussion

In this work, we used a state model to derive relations between incidence and mortality rates and the prevalence of undiagnosed and diagnosed persons in a chronic disease. The result is a two dimensional system of partial differential equations (PDEs) that forms a basis for estimating the incidence of the undiagnosed and diagnosed disease states from the prevalence of the associated states. In a simulation study and data from the *Health and Retirement Study* (HRS) we were able to show the accuracy and demonstrate the practical applicability of the method.

This method has several potential applications. First, it provides an approach to estimate the combined incidence for diabetes and similar conditions for which a large proportion of cases are undiagnosed and there is a lag in the identification of cases due to lack of symptoms, awareness, or health care access. Second, the methods also provide a way to determine the degree to which trends in incidence are biased by changing levels of detection by examining the ratio of diagnosed to undiagnosed incidence.

In an example, we have demonstrated the applicability of the modeling framework for a hypothetical chronic disease that has been discovered at a specific point in time, and has been diagnosed and treated thereafter. Apart from the hypothetical example, the analysis of the HRS data has proven applicability to real world data. Unfortunately, the uncertainty in the input data from HRS leads to relatively high uncertainty in the estimated incidence rates (Tables 2 and 3). As the HRS study has not been powered to accurately estimate the mortality of the study participants, the high uncertainty is a consequence of the study design. A more general analysis of how uncertainties and errors in the input data propagate into the results of the estimation, are subject of future work.

Our state model is an extension of the well-known illness-death model [15, 25], which has one additional state, *Undiagnosed*, which represents the subjects having contracted the disease but who are as yet undiagnosed. Using PDEs in the context of state models is not new [14, 26] and neither is taking into account undiagnosed diabetes [27]. However, the combination of both approaches is novel, and although our examples only considered non-communicable diseases, the model is potentially also applicable to some incurable infectious diseases, such as Hepatitis C or HIV, that have an asymptomatic preclinical phase [28].

The system of PDEs essentially has three advantages compared to other modelling techniques. First, the discretization errors using models with discrete time increments can be avoided. An example of these errors and the enormous impact they may have is demonstrated in [14], [Sect. 5]. Thus, the approach used here is more accurate than using discrete time models. The second advantage of PDEs lies in the fact that these equations are very well understood from the mathematical point of view. With very few assumptions on the smoothness of the right-hand side of the PDE, the existence and uniqueness of the solution is guaranteed [19]. Furthermore, there are a variety of freely available numerical routines to calculate the solution of PDEs. The third advantage is their flexibility: the new method may be applied to other chronic diseases as well, such as chronic kidney disease, osteoporosis, and cardiovascular disease. For each of these conditions, there exist modeling approaches including undiagnosed cases, which are important in health-economic models and screening [29, 30].

A drawback of the method is the restriction to *irreversible* chronic diseases. As shown in Fig. 1, it is assumed that there is no possibility to return to the *Normal* state. In case of diabetes, we know that this assumption is false, as bariatric surgery leads to remission in a large proportion of cases [31]. Even in the case of a modest intervention, like that received by the control condition of the *Look AHEAD Study*, 2 % had remission in the first year [32]. Thus, a small percentage of the incident cases may return to the *Normal* state [33, 34]. For diabetes, however, these cases are rare and have little impact on the population level that we are interested in. Other chronic diseases, like dementia do not have the possibility of remission at all. Thus, we consider the proposed methods useful for exploring how awareness for a disease and diagnostic possibilities may have an impact on the incidence of the disease. The full potential of the method is likely to become clear when time trends of the detection ratio of a chronic disease are studied.

In summary, we have developed a four-part compartment model with differential equations to estimate undiagnosed and diagnosed disease incidence and detection ratios for chronic diseases with common undiagnosed states. Future studies should validate our model using prospective, population-based studies, and surveillance systems.

## Conclusion

Based on the four-state compartment model we derived relations between the prevalences and the transition rates in terms of a system of partial differential equations. The partial differential equations provide insight into the epidemiology of undiagnosed chronic diseases. The applicability of the modeling framework has been demonstrated in a simulation study and in the analysis of the *Health and Retirement Study*.

## Additional file

Additional file 1
**Scripts for the statistical software R.** The zip-file contains the analysis for the simulation. For detailed instructions unzip the file and refer to the readme.txt file. (ZIP 3.01 Kb)
